# Bacterial xylanases: biology to biotechnology

**DOI:** 10.1007/s13205-016-0457-z

**Published:** 2016-06-30

**Authors:** Hillol Chakdar, Murugan Kumar, Kuppusamy Pandiyan, Arjun Singh, Karthikeyan Nanjappan, Prem Lal Kashyap, Alok Kumar Srivastava

**Affiliations:** 1 ICAR-National Bureau of Agriculturally Important Microorganisms (NBAIM), Kushmaur, Mau, Uttar Pradesh 275103 India; 2 ICAR-Indian Institute of Wheat and Barley Research (IIWBR), Karnal, Haryana India

**Keywords:** Bacteria, Xylanase, Thermostability, Alkali stability, Biotechnology

## Abstract

In this review, a comprehensive discussion exclusively on bacterial xylanases; their gene organization; different factors and conditions affecting enzyme yield and activity; and their commercial application have been deliberated in the light of recent research findings and extensive information mining. Improved understanding of biological properties and genetics of bacterial xylanase will enable exploitation of these enzymes for many more ingenious biotechnological and industrial applications.

## Introduction

Xylan is the second most abundant polysaccharide in nature present in both hard woods and annual plants. This homopolysaccharide is as abundant as cellulose accounting for approximately one-third of the renewable organic carbon sources on the earth (Kamble and Jadhav 2012). Structure of xylan varies among different plant species and its homopolymer backbone chain can be substituted with different side chain groups at various positions (Wang et al. 2014). Owing to its heterogeneity and complexity, complete hydrolysis of xylan requires variety of cooperatively acting enzymes collectively known as xylanases. Among various groups of microorganisms, bacteria and fungi are endowed with powerful xylanolytic machinaries. Xylanolytic microorganisms have been reported from various extreme environments, such as thermal springs (Bouacem et al. 2014), marines (Annamalai et al. 2009), Antarctic environments (Bradner et al. 1999), and soda lakes (Huang et al. 2015). Xylanases have wide range of industrial and biotechnological applications. Their commercial exploitation in the area of food (Harris and Ramalingam 2010), feed, and paper and pulp industries (Polizeli et al. 2005) are well documented. Recently, xylanases are also being used to increase the sugar recovery from agricultural residues for biofuel production (Gonçalves et al. 2015).

Due to huge industrial applications, a significant research effort has been devoted towards mining and characterization of xylanases. Initial focus had been on the fungal xylanases due to their high activity, but constraints faced during mass production and industrial applications, placed bacterial xylanases as a tough competitor in the industrial arena. Distribution, properties, genetics, and application of the bacterial xylanases have been discussed in this review to highlight their real potential as a promising source.

## Xylan and xylanases

Structurally xylan is a homopolymer of D-xylopyranose residues in β (1 → 4) linkages with a degree of polymerization ranging from 150 to 200. This backbone is substituted by some of the sugars and organic acids, such as arabinose, glucuronic acid, ferulic acid, etc. Xylans are broadly categorized into four major groups based on its substituents, viz., homoxylan, arabinoxylan, glucuronoxylan, and glucuronoarabinoxylan. Homoxylans contain xylose residues only, and can be either linear or branched (Sun et al. 2011). Arabinoxylans consist of a (1 → 4)-β-xylan main chain, but is substituted with α-arabinosyl residues. The β-(1 → 4)-linked d-xylopyranosyl residues are substituted with one α-(1 → 2)-linked 4-*O*-methyl-d-glucuronic acid in the case of glucuronoxylan; while in glucuronoarabinoxylans, the same backbone is linked to arabinofuranose and uronic acid (Gröndahl et al. 2003; Bergmans et al. 1996). The side chains determine the solubility, physical conformation, and reactivity of xylan molecule with other hemicellulosic components, and, hence, greatly influence the mode and extent of enzymatic cleavage.

Due to complexity in its structure, xylan needs different enzymes collectively termed as xylanases for its complete hydrolysis. Xylanases basically belong to hydrolase group of enzymes, precisely to glycoside hydrolases. Sequence-based glycoside hydrolase classification has placed xylanase in two families GH10 and GH11, but xylanases are also found in other glycoside hydrolase families, such as GH5, 7, 8, and 43. Plant, fungal, and bacterial enzymes comprise GH10 family, whereas GH11 family which is structurally unrelated includes only fungal and bacterial enzymes (Lafond et al. 2014). At least ten subfamilies of xylanases, some of which are restricted to fungi (xylanases Ia, Ib, Ic, II, IIIa, IIIb, and IV), and others to bacteria (A, B, C). GH10 family is composed of both endo-1,4-β-xylanases and endo-1,3-β-xylanases, but the majority being endo-1,4-β-xylanases with few endo-1,3-β-xylanases. They have greater catalytic versatility and can catalyze hydrolysis of even cellulose and aryl β-d-cellobiosides. In mixed linkage xylan, GH10 xylanases can attack on β-1,4-linkages that precede a β-1,3-linkage, but not the ones that immediately follow β-1,3-linkages. GH10 xylanases can attack β-1,3-linkages flanked on both sides by β-1,4-linkages. This family of xylanases can also tolerate replacement of one or two consecutive xylose residues by glucose residues in the substrate. Members of this family are capable of hydrolyzing the aryl β-glycosides of xylobiose and xylotriose at the aglyconic bond. Furthermore, these enzymes are highly active on short xylooligosaccharides, thereby indicating small substrate-binding sites (Pollet et al. 2010). Analyses of crystal structure, kinetic activity on xylooligosaccharides, and end products have indicated that family 10 xylanases typically have four-to-five substrate-binding sites. Most of the GH 10 xylanases typically have high molecular mass, low pI, and (α/β)_8_-barrel fold conformation (Teplitsky et al. 2000). GH11 members are monospecific, as they consist exclusively of true endo-β-1,4-xylanases that cleave internal β-1,4-xylosidic bonds. Their catalytic versatility is lower than GH10 members, and the products of their action can be further hydrolyzed by the family 10 enzymes. These are considered as “true xylanases”, because of their exclusive action on d-xylose containing substrates. Members of this family have low molecular mass, high pI, and a wide range of pH optima varying from 2 to 11. Like GH10 xylanases, these enzymes can hydrolyze the aryl β-glycosides of xylobiose and xylotriose at the aglyconic bond, but, unlike GH10, they cannot cleave cellulose or aryl β-d-cellobiosides. GH11 preferably cleaves un-substituted regions of the backbone, since they cannot attack the xylosidic linkage towards the non-reducing-end next to a branched xylose. Structure of GH11 enzymes principally consist of 3 β-pleaded sheets and one α-helix (Biely et al. 1997; Collins et al. 2005; Pollet et al. 2010). Some genera of fungi and bacteria produce more than one subfamily of xylanases. This means a possible duplication of family 11 xylanases might have occurred before the divergence between eubacteria and other domains (Sapag et al. 2002). GH5 family of xylanases is not as prevalent as of their GH10 and GH11 families but play an important role in complementing the action of GH10 and GH11 enzymes during depolymerization of glucuronoxylans in lignocellulosic fibres (Gallardo et al. 2010). GH8 xylanases have both high pI and molecular weight. Six enzymes from this family have been shown to have xylan degrading activity (Collins et al. 2012; Hong et al. 2014). GH8 xylanases have substrate specificity and action pattern similar to GH11 enzymes (Collins et al. 2006).

## Xylanolytic bacteria and their xylanases

Xylanases are produced by fungi, bacteria, yeast, marine algae, protozoa, snails, crustaceans, insect, seeds, etc. (Polizeli et al. 2005) Bacterial genera, such as *Bacillus*, *Cellulomonas*, *Micrococcus*, *Staphylococcus*, *Paenibacillus*, *Arthrobacter*, *Microbacterium*, *Pseudoxanthomonas,* and *Rhodothermus* have been reported to produce xylanases (Subramanian and Prema 2000; Beg et al. 2001; Gupta et al. 2001; Chapla et al. 2012). Among the actinomycetes group, *Streptomyces, Actinomadura*, *Nonomuraea*, etc. are commonly reported for xylanase production (Prakash et al. 2013).

Prevalence of the xylanolytic bacteria have been reported from most of the bacterial groups. Various kinds of xylanases with thermostability, cold adaptivity, or alkalo-stability have been mined and purified from diverse microorganisms, including extremophilic bacteria (Table 1). Among bacteria, *Bacillus* was found to be a potential source of xylanases, and a number of bacilli, such as *B. circulans*, *B. stearothermophilus*, *B. amyloliquefaciens*, *B. subtilis*, *B. pumilus,* and *B. halodurans* (Subramanian and Prema 2002; Thomas et al. 2014; Banka et al. 2014; Gupta et al. 2015) have been reported to have considerable xylanolytic activity. Thermostable xylanases active at temperature as high as 60–70 °C have been reported from *Bacillus* spp., *Stenotrophomonas maltophila*, *Rhodothermus marinus*, *Thermotoga* sp., *Clostridium thermocellum*, and *Streptomyces* sp. (Kumar and Satyanarayana 2013; Raj et al. 2013; Thomas et al. 2014). Although cold-adaptive xylanases are not very common, but bacteria, such as *Flavobacterium frigidarium* and *Clostridium* sp. PXLY1 (Akila and Chandra 2003; Humphry et al. 2001) have been reported to produce such xylanases. Bacteria have an advantage over fungi for xylanase production as pH optimum for bacterial xylanases are in neutral or alkaline range, whereas for fungal xylanases, it is in the acidic range. The low pH requirement for growth of fungi and production of fungal xylanases necessitates additional steps in the subsequent stages which make fungal xylanases less attractive. Firmicutes, such as *B. pumilus*, *B. halodurans,* and *Geobacillus thermoleovorans* (Verma and Satyanarayana 2012), and actinomycetes, such as *Streptomyces* sp. abd *Actinomadura* sp. (Luo et al. 2015; Taibi et al. 2012) have been reported as potential source of alkali stable xylanases. Xylanases with such unique features have found diverse application in various industries.

**Table 1:** Different groups of bacteria producing diverse xylanases

S. no.	Name of the organism	Substrate used	Optimum conditions for xylanase activity				Purification of xylanase				References
			Temp (°C)	pH	*K*_m_ (mg/ml)	*V*_max_ (µmol/mg/min)	Methods	Specific activity (U/mg)	Purification (fold)	Recovery (%)	
Mesophilic											
1.	*Sorangium cellulosum* So9733-1	OSX	30	7.0	38.13	10.69	Dialysis and concentration after Ni-affinity	4.11	4.03	43.84	Wang et al. (2012)
2.	*Bacillus* sp. SN5	BeX	40	7.0	0.6	114	Ni-affinity chromatography	104.7	–	–	Bai et al. (2012)
3.	*Paenibacillus xylanilyticus KJ*-*03*	BiX	40	7.4	–	–	Ni-affinity chromatography	33	7.5	58	Park et al. (2013)
4.	*Bacillus* sp. SV-34S	BiX	50	6.5	3.7	133.33 IU/ml	(NH_4_)_2_SO_4_ precipitation	2803.1	10.62	88.0	Mittal et al. (2012)
							Carboxymethyl-sephadex C-50	3417.2	12.94	13.44	
5.	*Streptomyces* sp. 7b	WB	50	6	–	–	(NH_4_)_2_SO_4_ precipitation (50-75 %)	79.43	2.46	47.80	Bajaj and Singh (2010)
							CM-sephadex	183.50	5.68	43.41	
6.	*Burkholderia* sp. DMAX	BiX	50	8.6	12.75	165	(NH_4_)_2_SO_4_ precipitation (30-70 %)		3.21	48	Mohana et al. (2008)
Thermophilic											
7.	*Anoxybacillus favithermus *TWXYL3	OSX	65	6 and 8	–	–	HiPrep 26/60 Sephacryl S-200	2.2	27.5	–	Ellis and Magnuson (2012)
8.	*Bacillus* sp. GRE7	OSX	70	7.0	2.23	296.8 IU/mg	(NH_4_)_2_SO_4_ precipitation (40-80 %)	191.1	3.9	71	Kiddinamoorthy et al. (2008)
							DEAE- cellulose	582.9	11.9	48	
							Sephadex G-75	1392.6	28.5	27	
9.	*Streptomyces thermovulgaris* TITSR1948	BeX	65	6.5	0.76	303U/mg	DEAE- Toyopearl	27	3.70	–	Boonchuay et al. (2016)
							Toyopearl HW-55	110	15.0	–	
10.	*Arthrobacter* sp.	WB	100	9.0	0.9	3571	(NH_4_)_2_SO_4_ precipitation	162	2	74	Khandeparkar and Bhosle (2006)
							Sephadex G-200	282.6	3.5	62	
							DEAE- sepharose FF	444.2	5.5	49	
							CM-sepharose FF	1697.7	21	14	
11.	*Geobacillus thermoleovorans*	BiX	80	8.5	2.6	31.2	Ni^2+^-NTA agarose resins	10.2	–	–	Verma and Satyanarayana (2012)
12.	*Stentrophomonas maltophilia*	WB	80	9.0	–	–	(NH_4_)_2_SO_4_ precipitation	82.40	1.43	57.11	Raj et al. (2013)
							Sephadex G-100	141.11	2.45	37.36	
							DEAE-cellulose	313.38	5.43	19.18	
13.	*Thermotoga thermarum*	BeX	80	6.0	1.8	769	Heat treatment	80	2.9	93.7	Shi et al. (2014)
							Ni-affinity chromatography	192	6.9	82.3	
14.	*Acidothermus cellulolyticus* 11B	OSX	90	6.0	0.53	350	Heat-treated extract	15.6	4.1	88	Barabote et al. (2010)
							Hydroxyapatite	376	97.7	12	
Marine											
15.	*Bacillus* sp.	OSX	55	9.0	–	–	(NH_4_)_2_SO_4_ precipitation (60 %)	31.27	2.6	55	Annamalai et al. (2009)
							DEAE-cellulose	53.0	1.69	34	
16.	*Arthrobacter* sp. MTCC6915	SD	60	9.0	–	–	(NH_4_)_2_SO_4_ precipitation	453.6 U/ml	–	–	Murugan et al. (2011)
17.	*Glaceocola mesophila* KMM 241	BeX	30	7.0	1.22	98.31	(NH_4_)_2_SO_4_ precipitation Dialysis Ni^2+^-NTA agarose resins	77	9.1	17.4	Guo et al. (2009)
18.	*Vibrio sp.* XY-214	β -1,3 xylan	37	7.0	–	–	(NH_4_)_2_SO_4_ precipitation Dialysis Q Sepharose FF Ether-Toyopearl 650S Superdex 200	9.9	83	24	Araki et al. (1999)
19.	*Alcaligenes* sp. XY-234	β -1,3 xylan from *Caulerapa racemosa*	40	7.5	0.40	–	(NH_4_)_2_SO_4_ precipitation Dialysis Q Sepharose FF Ether-Toyopearl 650S Superdex 200 Hydroxyapatite MonoQ		292	9	Araki et al. (1998)

## Comparison of bacterial and fungal xylanases

Majority of the bacterial xylanases belong to GH10 family, whereas fungal xylanases majorly belong to GH11 family (Liu et al. 2011). Endoxylanases from bacteria and fungi display exclusively single subunit protein structures with molecular weight (MW) ranging from 8.5 to 85 kDa and their isoelectric point (pI) values between 4.0 and 10.3 and most of them show glycosylation (Polizeli et al. 2005). In-silico structural comparison of bacterial and fungal xylanases shows difference in their secondary structure especially at the loop areas. *Bacillus circulans* and *B. subtilis* mainly have beta sheet structure, while fungal xylanase, especially *Aspergillus niger,* have alpha helices and also have difference in various loop regions though both belonged to class GH11. A common thing found in both fungal and bacterial xylanases is that the nucleophile and proton donor are always the Glutamic acid, though their position may change (Mathur et al. 2015). Fungal xylanases are produced simultaneously with cellulase that increases length of downstream processing; while in bacteria, xylanase are produced alone mostly, thereby reducing the downstream process time. Low pH requirement for growth of fungi and production of cellulase necessitates additional steps in downstream processing which make fungal xylanase less attractive (Subramanian and Prema 2002).

## Genetics of bacterial xylanases

**Fig. 1:**
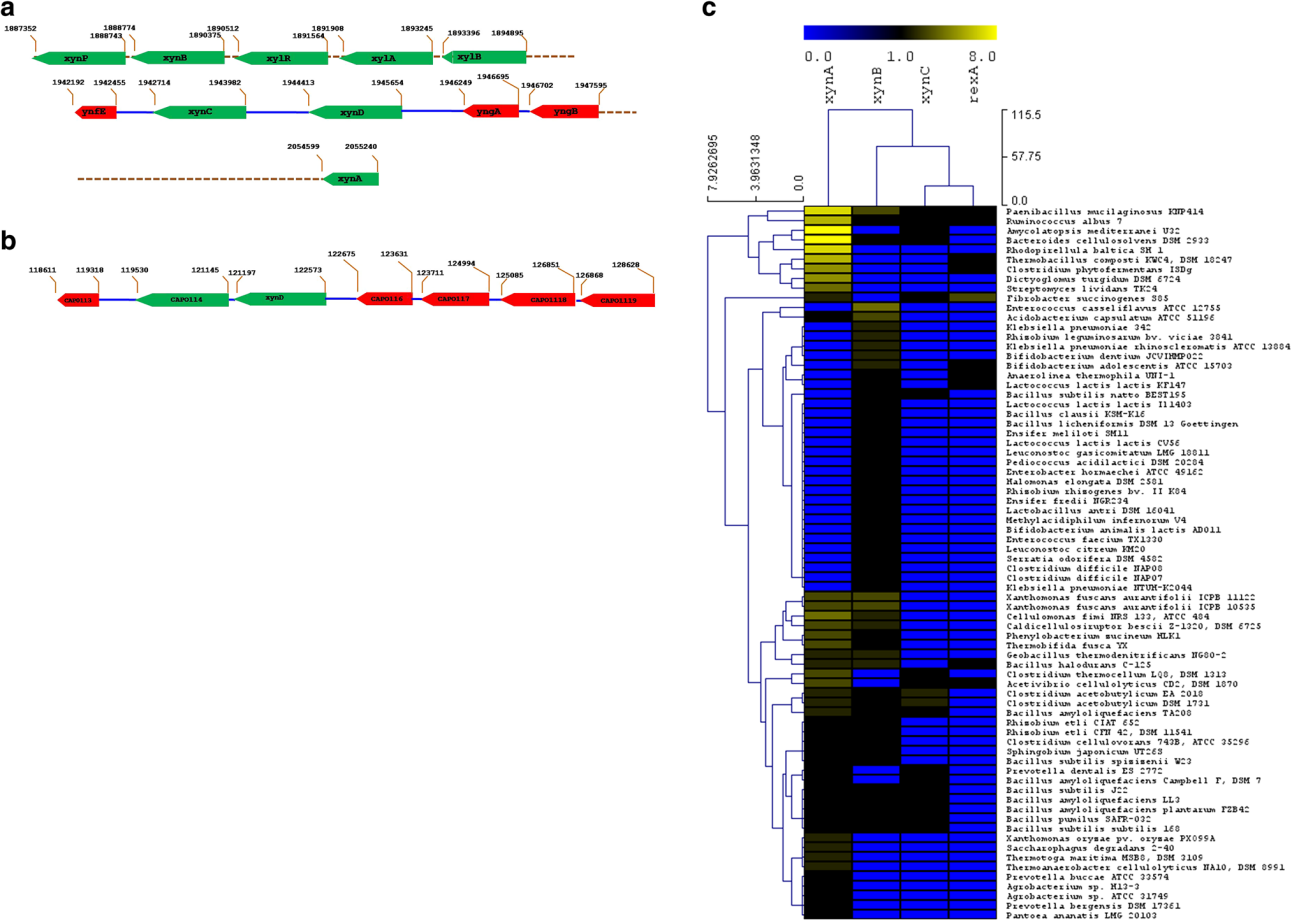
**a** Organization of xylanase genes on chromosome of *B. subtilis* str 168. **b** Organization of xylanase gene on plasmid of *C. acetobutylicum* ATCC824. **c** Diversity of xylanolytic bacteria based on composition and copy number of xylanase genes (clustering was performed based on weighted pair average and Manhattan distances using Multi-Experiment Viewer)

*Bacillus subtilis* and *Clostridium acetobutylicum* are two well-studied xylanolytic firmicutes. Whole genome sequencing of *B. subtilis* str 168 has provided in sight of the organization of the genes-coding xylanolytic enzymes and transporters for the utilization of xyloses. The genes-coding xylanolytic enzymes of *B. subtilis* str 168 are located in the chromosomal DNA. Four major genes, viz., *xyn*A (1241 bp), *xyn*B (1601 bp), *xyn*C (641 bp), and *xyn*D (1241 bp) are reported (Fig. 1a). *xyn*A encodes for endo-1,4 beta-xylanase (GH11 family) and depolymerizes xylan to produce methyl glucurono xylotetraose and xylooligosaccharides. *xyn*B codes for β-xylosidase, while *xyn*C encodes GH30 family endoxylanase (glucuronoxylanases). Generation of a series of aldouronates with an increasing number of xylose residues and a single methylated glucuronoxylan (MeG) linked-1, 2 to penultimate xylose residue from reducing-end is a characteristic of XynC. *xyn*D encodes arabinoxylan arabinofuranohydrolase that catalyzes the release of free arabinose from methyl galacturono xylans. XynD has higher activity on arabinoxylans compared to oligo-arabinoxylosides. β-xylanase is synthesized constitutively and in contrast to many other extracellular enzymes, it is synthezised mostly during exponential phase. On the other hand, β-xylosidase synthesis has been found to be strongly induced by xylose and xylan (Lindner et al. 1994). Xylose induction for the genes of the *xyn*CB and *xyl*AB operons had been described previously by Gartner et al. (1988) and Hastrup (1988). *xyl*A (1337 bp) encodes for xylose isomerase and *xyl*B codes for xylulose kinase. Upstream to the *xyn*B, another gene *xyn*P (1392 bp) encodes for sugar (glycoside-pentoside-hexuronide) transporter protein. The genes of the *xyn*CB and *xylA*B (involved in xylose utilization) operons are controlled by the transcriptional regulator encoded by *xyl*R (1053 bp). Expression of *xyl*AB is negatively regulated at the transcriptional level by the regulator XylR.

In case of *Clostridium acetobutylicum* ATCC 824, the xylanase encoding genes are located both on plasmid and chromosome. The organization of xylanase encoding genes on plasmid is presented in Fig. 1b. Like *B. subtilis*, *C. acetobutylicum* also have *xyn*B (957 bp) and *xyn*D (1376 bp) genes. *C. acetobutylicum* contains at least two homologous copies of *xyn*C (CAP_0118, 1766 bp and CAP_0119, 1758 bp). *xyn*D encodes GH43 endo-1,4 beta-xylanase, while *xyn*A encodes a GH35 endoxylanase. In addition, *C. acetobutylicum* encodes (CAP_0114, 1625 bp) another GH35 endoxylanase which also includes merged domains for arabinoxylan arabinofurano hydrolase, β-xylosidase, and α-l-arabinofuranosidase. The plasmid harbours a 1283 bp gene (CAP_0117) for β-xylosidase. *xyn*D (CA_C 3452) encoding another GH43 hydrolase is also located on chromosome. Upstream to this *xynD* gene, *xyn*T (1377 bp) encodes a xyloside transporter protein. In *C. acetobutylicum*, the xylose utilization pathway also includes a xylulokinase (XylB, CAC2612), a xylose proton-symporter (XylT, CAC1345), and a transcriptional regulator (XylR, CAC3673). *xyn*D and *xyn*T along with *xyl* genes are under transcriptional control of XylR. Bacteria are endowed with varying xylanolytic activity with distinct properties due to diversity in their xylanase genes. Table 2 presents list of genes and their coded products which have been thoroughly characterized at proteomic level, and protein models are available at public databases. However, the differential ability of the bacteria to degrade xylan with varying efficiency and distinct mode of action also depend on the composition and organization of xylanase genes. Global distribution of gene copy number among the xylan degrading gene cassette represented by four genes encoding endo-1,4-beta-xylanase (*xyn*A), xylan 1,4-beta-xylosidase (*xyn*B), glucuronoarabinoxylan endo-1,4-beta-xylanase (*xyn*C), and oligosaccharide reducing-end xylanases (*rex*A). Whole genome sequence data of 74 xylanolytic bacteria selected from diverse ecological niche, such as soil, rumen, human gut etc., that are available at JGI (Joint Genome Institute) was used to study the xylan degrading gene cassette. The abundance of xylan degrading genes in terms of gene copy number among the selected xylanolyitc bacteria was used to create dual dendrogram. Among the four genes, *xyn*A was most widely distributed, whereas *rex*A was very rare among the isolates. Presence and high abundance of particular xylan degrading genes help in natural selection of particular bacteria in a specialized ecological niche. For instance, cattle rumen-associated cellulo-xylanolytic bacterium *Fibrobacter succinogenes* S85 (Fig. 1c) has the highest copy number of *rex*A gene that endowed the bacterium to synthesise xylo-oligosachharide which supports the growth of other bacteria in rumen. *Paenibacillus mucilaginosus* KNP414 and *Ruminococcus albus* 7 were having the highest copy number of all xylan degrading gene and could be very useful for complete structural degradation of hemicellulosic biomass. Knowledge of relative gene copy number and gene composition through whole genome survey could play a vital role in selection of ecological niches for isolation experiments and further selection of bacterial group for mining novel xylanolytic bacteria.

**Table 2:** Xylanase genes and their products

Source	Gene	Product	Product length (a.a)	Enzyme class	MW (kDa)	PDB ID of protein model
*Bacillus circulans*	*xlnA*	Xylanase	185	Hydrolase (GH11)	20.761	1BCX
*Dictyoglomus thermophilum*	*xynB*	Beta-1,4-xylanase	199	Hydrolase (GH11)	44.813	1F5J
*Pseudoalteromonas haloplanktis*	*xyl*	Endo-1,4-beta-xylanase	405	Hydrolase (GH8)	46.024	1H14
*Streptomyces* sp. S38	*xyl1*	Endo-1,4-beta-xylanase	190	Hydrolase (GH11)	41.194	1HIX
*Thermopolyspora flexuosa*	*xyn11A*	Endoxylanase	197	Hydrolase (GH11)	22.702	1M4W
*Bacillus circulans*	*xlnA*	Xylanase	185	Glycosidase (GH11)	20.505	1XNB
*Bacillus circulans*	*xlnA*	Xylanase	185	Glycosidase (GH11)	20.414	1XNC
*Bacillus circulans*	*xlnA*	Endo-1,4-beta-xylanase	185	Hydrolase (GH11)	20.393	2BVV
*Bacillus* sp.	*xynJ*	xylanase J	354	Hydrolase (GH11)	79.794	2DCJ
*Escherichia coli*	*xyn11*	GH11 Xylanase	216	Hydrolase (GH11)	25.270	2VUL
*Bacillus subtilis*	*xynD*	Arabinoxylan arabinofuranohydrolase	487	Hydrolase (GH43)	53.119	3C7O
*Bacteroides thetaiotaomicron* VPI-5482	*BT_2895*	Endo-1,4-beta-xylanase	306	Hydrolase (GH43)	68.880	3KST
*Bacillus circulans*	*xlnA*	Endo-1,4-beta-xylanase	182	Hydrolase (GH11)	59.975	3LB9
*Thermopolyspora flexuosa*	*xyn11A*	Endo-1,4-beta-xylanase	193	Hydrolase (GH11)	21.625	3MF6
*Bacillus circulans*	*xlnA*	Endo-1,4-beta-xylanase	185	Hydrolase (GH11)	81.864	3VZJ
*Bacillus circulans*	*xlnA*	Endo-1,4-beta-xylanase	185	Hydrolase (GH11)	41.040	3VZK
*Bacillus circulans*	*xlnA*	Endo-1,4-beta-xylanase	185	Hydrolase (GH11)	82.020	3VZL
*Bacillus circulans*	*xlnA*	Endo-1,4-beta-xylanase	185	Hydrolase (GH11)	41.673	3VZN
*Bacillus circulans*	*xlnA*	Endo-1,4-beta-xylanase	185	Hydrolase (GH11)	20.893	3VZO
*Thermobifida fusca*	*xyl11*	Endo-1,4-beta-xylanase	201	Hydrolase (GH11)	22.845	3ZSE
*Caldicellulosiruptor bescii*	Athe_0185	Endo-1,4-beta-xylanase	345	Hydrolase (GH10)	41.682	4L4P

## Cloning and expression of xylanase genes

For commercial level production of xylanase, it is necessary to select bacteria which can overproduce xylanase. Recombinant DNA technology is one of the important techniques which provide ways to enhance enzyme production. Xylanase genes from different bacteria have been cloned into suitable hosts for overexpression (Kulkarni et al. 1999). Two xylanase genes (*xynA* and *xynB*) from a cellulolytic bacterium *Ruminococcus flavefaciens*17 were cloned and expressed in a heterologous host *E*. *coli* (Flint et al. 1991). Xylanase gene has also been cloned from an extreme thermophilic bacterium *Dictyoglomusthermophilum* Rt46B.1, employing a genomic DNA library screening approach. Sequencing the subclone is shown to contain a single complete open reading frame coding for a single-domain xylanase, *XynA*, with a putative length of 352 amino acids (Gibbs et al. 1995). A xylanase gene of 1383 bp coding for 460 amino acids with a molecular mass of 51.3 kDa was cloned from a marine bacterium *Vibrio* sp. strain XY-214 and expressed in *E*. *coli* DH5α. The transformant enzyme hydrolyzed β-1,3-xylan to produce several xylooligosaccharides (Araki et al. 2000). Two genes *XynA-* and *XynB-*encoding xylanases from *Paenibacillus* sp. KCTC 8848P were cloned and expressed in *Escherichia coli*. The structural gene of *XynA* 636 bp, encoded a protein of 212 amino acids, while the *XynB* gene consisted of 951 bp open reading frame for a protein of 317 amino acids. The xylanases of *E. coli* transformants were released into the extracellular culture fluid in the absence of xylan (Lee et al. 2000). A xylanase gene of 642 bp length was cloned from *Bacillus subtilis* B10 isolated for degumming of ramie blast fibres. Xylanase gene was expressed and xylanase activity was measured. The xylanase distribution in extracellular, intracellular, and periplasmic fractions was about 22.4, 28.0, and 49.6 %, respectively (Huang et al. 2006). An alkalistable and thermostable xylanase gene *Myxl* was cloned from a metagenomic library constructed from DNA extracted from the compost-soil. Sequence analysis of the clone revealed a xylanase gene of 1077 bp. The deduced protein sequence (358 amino acids) displayed homology with glycosyl hydrolase (GH) family 11 xylanases. The gene was subcloned into pET28a vector and expressed in *E. coli* BL21 (DE3). The recombinant xylanase (rMxyl) exhibited activity over a broad range of pH and temperature with optima at pH 9.0 and 80 °C. The recombinant xylanase is highly thermostable having T1/2 of 2 h at 80 °C and 15 min at 90 °C (Verma et al. 2013a, b). A xylanase gene was isolated from *Bacillus brevis* and expressed in *E*.*coli* BL21. The recombinant xylanase was predominantly secreted to culture medium and showed mesophilic nature (optimum activity at 55 °C and pH 7.0). The cell free culture medium exhibited 30 IU/ml xylanse activity. The enzyme did not show any cellulose activity and was active under wide range of temperature and pH (Goswami et al. 2014).

## Factors affecting xylanase yield and activity

Although an efficient xylanase producing microorganism is a prerequisite for industrial fermentation of xylanases, the yield during fermentation process is a function of factors, such as nutritional requirements and environmental conditions. Among them, nutritional factors, the carbon sources present in the media, are the most important factors for xylanase production. A number of substrates, such as wheat bran, rice bran, soy meal, sugarcane bagasse, etc., have been found to be suitable substrates for xylanase production (Kumar et al. 2009; Raj et al. 2013). Murugan et al. (2011) reported xylanase production (117 U/ml) using saw dust as substrate for *Arthrobacter* sp. MTCC6915 under solid-state fermentation (SSF), while Sepahy et al. (2011) reported xylanase activity (249.308 IU/ml) in *Bacillus mojavensis* AG137 using oat bran under submerged fermentation. *Bacillus subtilis* ASH have been reported to produce xylanase with wheat bran, wheat straw, rice husk, saw dust, gram bran, groundnut, and maize bran under SSF (Sanghi et al. 2008). Boonchuay et al. (2016) reported xylanase production from *Streptomyces thermovulgaris* TISTR1948 using corn cob. Wheat bran has been reported to be the best substrate for the maximum production of xylanase enzyme (Sanghi et al. 2008; Kapoor et al. 2008; Nagar et al. 2010; Kumar et al. 2013). The reason might be due to the fact that it contains 54 % carbohydrates (pentoses and hexoses), 14 % protein, minerals, amino acids, and vitamins (El-Sharnouby et al. 2012), which supports the growth of the bacterium and hence xylanase production. In addition, substrate accessibility, rate, and quantity of release of the xylooligosaacharides, their chemical nature, and quantity of xylose released also influence the production of xylanases during fermentation process. Guha et al. (2013) reported sugarcane bagasse as best inducer for xylanase production from *Bacillus* sp. Yeast extract, beef extract, peptone, etc. can serve as good nitrogen source for yielding xylanase. Enhanced levels of xylanase from *Streptomyces cyaneus* SN32, *Bacillus* sp., *Streptomyces* sp., *Streptomyces* sp. CA24 have been reported using yeast extract, peptone, and beef extract as organic nitrogen source (Porsuk et al. 2013). Apart from the substrates, the method of fermentation influences the number of isoforms produced by bacterium. For example, wheat bran induced six isoforms under SSF, but only one under submerged fermentation in *Simplicillium obclavatum* MTCC 9604. Surprisingly, three among six isoforms were of similar molecular weight, but with different pI which helps them to achieve effective hydrolysis of xylan in wider pH range (Roy et al. 2013). In the case of *Providencia* sp. strain X1, it produced seven isoforms with wheat bran, but only two with birchwood xylan (Raj et al. 2013). The need for multiple forms of xylanase could be due to the complexity in lignocellulosic biomass to achieve maximum sugar yield, and they could arise from post-translational modification of a gene product, such as differential glycosylation or proteolysis (Sharma and Chadha 2011). Besides nutritional components, other bioprocess parameters, such as pH of the medium, incubation temperature, agitation, initial load of inoculum, etc., also influence the xylanase production during fermentation process. Among these factors, pH and temperature determine the activity and stability of enzyme. Xylanases obtained from bacterial sources are known to be active and stable in wide range of pH and temperature. Xylanase enzyme exhibit its optimal activity wide ranges of incubation conditions, such as temperature from 30 to 60 °C, pH from 5.0 to 9.0. In many cases, the optimum conditions for production are differ from its activity. For example, the temperature for xylanse production was 30 and 37 °C, but it showed its optimal activity at 60 and 55 °C, respectively (Murugan et al. 2011; Sepahy et al. 2011). Inoculum size of 1 % and agitation rate of 120 or 200 proved to be the suitable parameters for maximum production of xylanase under submerged fermentation (Mittal et al. 2012; Raj et al. 2013; Kaur et al. 2016). Presence of carboxymethyl cellulose, dextrose, peptone, and beef extract supported the maximum xylanase production in the range from 161.4 to 176.4 U/ml (Murugan et al. 2011). Table 3 elaborates factors, such as substrate, pH, and temperature, affecting the activity of some selected bacterial xylanases.

**Table 3:** List of bacterial xylanases and factors affecting their activity

Sl. no.	Bacteria	Activity	Substrate	Reaction conditions	Reference
1	*Actinomadura* sp. Strain Cpt20	51.06 U/mg	Oat spelt xylan & Beech wood xylan	80 °C; pH 10.0	Taibi et al. (2012)
2	*Anoxybacillus* *flavithermus* WL	2.2 U/mg	Oat spelt Xylan	65 °C; pH 7.0	Ellis and Magnuson (2012)
3	*Bacillus brevis*	4380 U/mg	Agro-waste like wheat straw	55 °C; pH 7.0	Goswami et al. (2014)
4	*Bacillus halodurans*	69 U/ml	Cane molasses	80 °C	Kumar and Satyanarayana (2013)
5	*Bacillus pumilus* SSP-34	1723 U/mg	Oat spelts xylan	50 °C; pH 6.0	Subramaniyan (2012)
6	*Bacillus pumilus* SV-205	7382.7 IU/ml	Wheat bran	60 °C; pH 10.0	Nagar et al. (2012)
7	*Bacillus subtilus* BS05	439 IU^−1^	Sugarcane baggase	50 °C; pH 5.0	Irfan et al. (2012)
8	*Gracilibacillus* sp.TSCPVG	1667 U/mg	Birchwood xylan	pH 7.5	Poosarla and Chandra (2014)
9	*Jonesia denitrificans* BN-13	77 U/mg	Birchwood xylan	50 °C; pH 7.0	Boucherba et al. (2014)
10	*Kluyvera* sp. OM3	5.12U/ml	Birchwood xylan	70 °C; pH 8.0	Xin and He (2013)
11	*Paenibacillus* sp. NF1	3081.05 IU/mg	Oat spelt xylan	60 °C; pH 6.0.	Zheng et al. (2014)
12	*Paenibacillus macerans *IIPSP3	4170 U/mg	Beechwood xylan	60 °C; pH 4.5	Dheeran et al. (2012)
13	*Paenibacillus* sp. N1	24.60 IU/ml	Reese medium	50 °C; pH 9.0	Pathania et al. (2012)
14	*Providencia* sp. strain XI	36.3 IU/ml	Wheat bran	60 °C; pH 9.0	Raj et al. (2013)
15	*Stenotrophomonas* *maltophilia*	26.4 IU/ml	Wheat bran	80 °C; pH 9.0	Raj et al. (2013)
16	*Thermoanaerobacterium* *calidifontis* sp.nov.	16.2 U/ml	Oat spelt xylan	50–55 °C; pH 7.0	Shang et al. (2013)

## Application of xylanases

Most of the bacterial xylanases are alkali- and/or thermostable and could be free of cellulose activity. These features make them ideal candidate for industrial applications. A number of bacterial xylanases, including their production technology, have been patented for various applications, and have been marketed for numerous commercial applications worldwide (Table 4). Some of the major applications of bacterial xylanases are discussed below.

**Table 4:** Commercial products of bacterial xylanases

S. no	Product	Company	Source	Application
1	“Propan BXC”	Aumgene Biosciences, India	Bacteria	Bakery
2	“Bleachzyme F”	Biocon India, Bangalore	n. c.	Bleaching of pulp
3	“Pulpzyme HA, HB,HC”	Novozymes, Denmark	*Bacillus* sp.	Cellulose and paper industry
4	“Panzea”	Novozyme, Denmark	*B. licheniformis*	Bakery
5	Luminase	Verenium	Bacteria from thermal spring	Bleaching of pulp
6	Belfeed B1100	Agrimex, Belgium	Bacteria	Feed additive
7	Nutri Xylanase Enzyme	Ultra Biologics Inc., USA	*Bacillus subtilis*	Feed additive
8	Bacterial Xylanase XBK BX9	Leveking, China	Bacteria	Bakery
9	Xylanase(bacterial)	Biovet JSC, Bulgaria	*Bacillus subtilis*	Bakery, pulp and paper industries, feed additive
10	Cartazyme HS	Sandoz, UK	*Thermomonospora fusca*	

### Paper and pulp industry

Xylanase enzymes to be used for biobleaching of pulps must be active at high temperatures and alkaline pH, and must not contain cellulolytic enzymes to preserve the cellulose fibres (Polizeli et al. 2005). Any cellulase activity will have serious economic implications in terms of cellulose loss, degraded pulp quality, and increased effluent treatment cost. Xylanases from various microbes have been screened for their efficiency in enzyme aided bleaching of wood pulp. Many bacterial xylanases binds to both cellulose and insoluble xylan, but the enzyme has activity against only xylan. Thus, fungal-based xylanases have a lesser degree of hemicellulose removal for a given charge than those of bacterial-based enzymes. In comparison to fungal xylanases, bacterial enzymes tend to produce a significant kappa reduction. The fungal enzymes are thought not to exhibit the same level of tenacity toward carbohydrate binding domains; hence, they are less aggressive than bacterial-based enzymes (Wong et al. 1988). Use of xylanase can significantly reduce the amount of chlorine used for bleaching of pulp (Viikari et al. 1986; Paice et al. 1988; Clarke et al.^ 1991^; Jeffries et al. 1992) Biobleaching of eucalyptus kraft pulp using xylanase from *B. pumilus* ASH5 resulted in reduction of chlorine and chlorine dioxide consumption by 20 and 10 %, respectively, besides improved brightness and whiteness of pulp (Battan et al. 2007). Chapla et al. (2012) reported similar result using cellulose free thermostable xylanase from *Paenibacillus* sp. ASCD2 in prebleaching of eucalyptus kraft pulp.

### Deinking of waste paper

One of the important steps in recycling of waste paper is the deinking process which involves dislodgement of ink particles from paper. Conventionally, large amount of chlorine, chlorine-based derivatives, sodium hydroxide, sodium carbonate, sodium silicate, hydrogen peroxide, hypochlorites, and chelating agents have been used which resulted in hazardous effluent disposal problem (Maity et al. 2012). Recently, xylanase and laccase enzymes have been reported to remove ink from effluent generated from paper and pulp industries (Dhiman et al. 2014; Chandra and Singh 2012). An attempt has been made to deink old newsprint using the combined applications of xylanase and laccase. Combined action resulted in significant increase in brightness (11.8 %), whiteness (39 %) and physical properties like breaking length (34.8 %), burst factor (2.77 %), and tear factor (2.4 %). Synergism between xylanase and laccase during co-treatment would have positively influenced physical properties, whereas chemical treatment for deinking would has deteriorated freeness and strength of the recycled paper (Gupta et al. 2015). Kumar and Satyanarayana (2014) tried different combinations of commercial cellulase and xylanase from *Bacillus halodurans*TSEV1. They found that xylanase dose of 1.2 U/mg along with commercial cellulase at 1.2 U/mg was efficient in ink removal.

### Animal feeds

Xylanases are used to improve digestibility of animal feed. Low viscosity in some cereals, such as maize and sorghum, is attributed to their arabinoxylan content which renders them unsuitable as feed. Xylanase addition to feeds improves digestion in the initial part of digestive tract resulting in better energy use (Harris and Ramalingam 2010). Clarkson et al. (1999) reported better digestibility of cereal feeds for poultry resulting in reduction in feed conversion ratio (FCR) using endoxylanases from *Acidothermus cellulolyticus*. Babalola et al. (2006) observed improved apparent nitrogen and fiber absorption as well as feed transit time. In general, a combination of different enzymes is supplemented with animal feed to achieve favorable results. A wheat based diet supplemented with combination of phytase, xylanase, and β-glucanase improved the performance of broiler chickens. Improved performance is attributed to reduced digesta viscosity, increased apparent metabolisable energy, reduced relative weight, and length of small intestine (Wu et al. 2004). A number of bacterial xylanases have been commercially available for uses as feed additive.

### Bakery industry

Presence of hemicelluloses, such as arabinoxylans, in wheat, the key raw material of baking industry, pose serious constraints in making of good quality dough. The ability of xylanases to solubilize water unextractable arabinoxylan (WU-AX) and its low activity on water extractable arabinoxylan (WE-AX) results in better performance during bread making as a result of its solubilized arabinoxylan (Courtin and Delcour 2002). Bacterial xylanases like GH11 endoxylanases from *B. subtilis* solubilize WU-AX, in particular, increases the viscosity of dough and has a negative effect on gluten agglomeration (Butt et al. 2008). Xylanase transforms water insoluble hemicellulose into soluble form, which binds water in the dough, therefore, decreasing the dough firmness, increasing volume, and creating finer and more uniform crumbs (Butt et al. 2008).

### Biofuel production

Conversion of abundant lignocellulosic plant biomass into biofuels presents a viable option for improving energy security and reducing greenhouse emissions (Kumar et al. 2009). One of the major limitations is the recalcitrant nature of the plant cell wall, which is composed mostly of lignocellulosic materials, such as lignin, cellulose, and hemicelluloses. Xylanase, together with other hydrolytic enzymes, such as cellulases and laccases can be used for the generation of biofuels, such as ethanol, from lignocellulosic biomass. Bacteria are rich source of thermostable xylanases and are generally preferred for lignocellulose hydrolysis. Robust thermostability of bacterial xylanases make them well suited for the harsh processing conditions required during deconstruction of lignocellulose to fermentable products. Bhalla et al. (2015) reported higher hydrolytic conversion of Birchwood xylan (68.9 %) with *Geobacillus* sp. strain WSCUF1 as compared to commercial enzymes, such as Celic HTec2 (49.4 %) and Accelerase XY (28.92 %), at 70 °C. The supplementation of xylanases during enzymatic saccharification increased the availability of reducing sugars which ultimately results in high bioethanol production.

### Pharmaceutical industry

Hydrolytic products of xylan known as xylooligosaccharides (XOs) are mixtures of oligosaccharides formed by xylose residues linked through β-1,4 linkages. On the basis of number of xylose residues, they are known as xylobiose, xylotriose, xylotetrose, and so on. Xylobiose is ideal XO for food application as prebiotic. XOS are produced from xylan containing lignocellulosic biomass (LCM) by chemical, enzymatic (Katapodis et al. 2002), and combination of both the methods (Kokubo and Ikemizu 2004). For the production of XOs, the enzyme complex should have low exoxylanase or β-xylosidase activity, to prevent the production of high amounts of xylose, which has inhibitory effects on XOs production (Liu and Liu 2008; Boonchuay and Chaiyaso 2012; Verma et al. 2013a, b; Jain et al. 2015). XOs have a huge commercial market due to their health promoting properties. Besides chemical processes, enzymatic hydrolysis of xylans using endoxylanases from bacterial genera, such as *Bacillus*, *Streptomyces*, *Rhodothermus*, *Thermobifida,* etc., are being used. Detailed genomic information has provided opportunities for engineering bacterial strains to produce desired XOs. e.g. Rhee et al. (2014) engineered *B. subtilis* str 168 to produce acidic XOs.

## Future perspectives

**Fig. 2:**
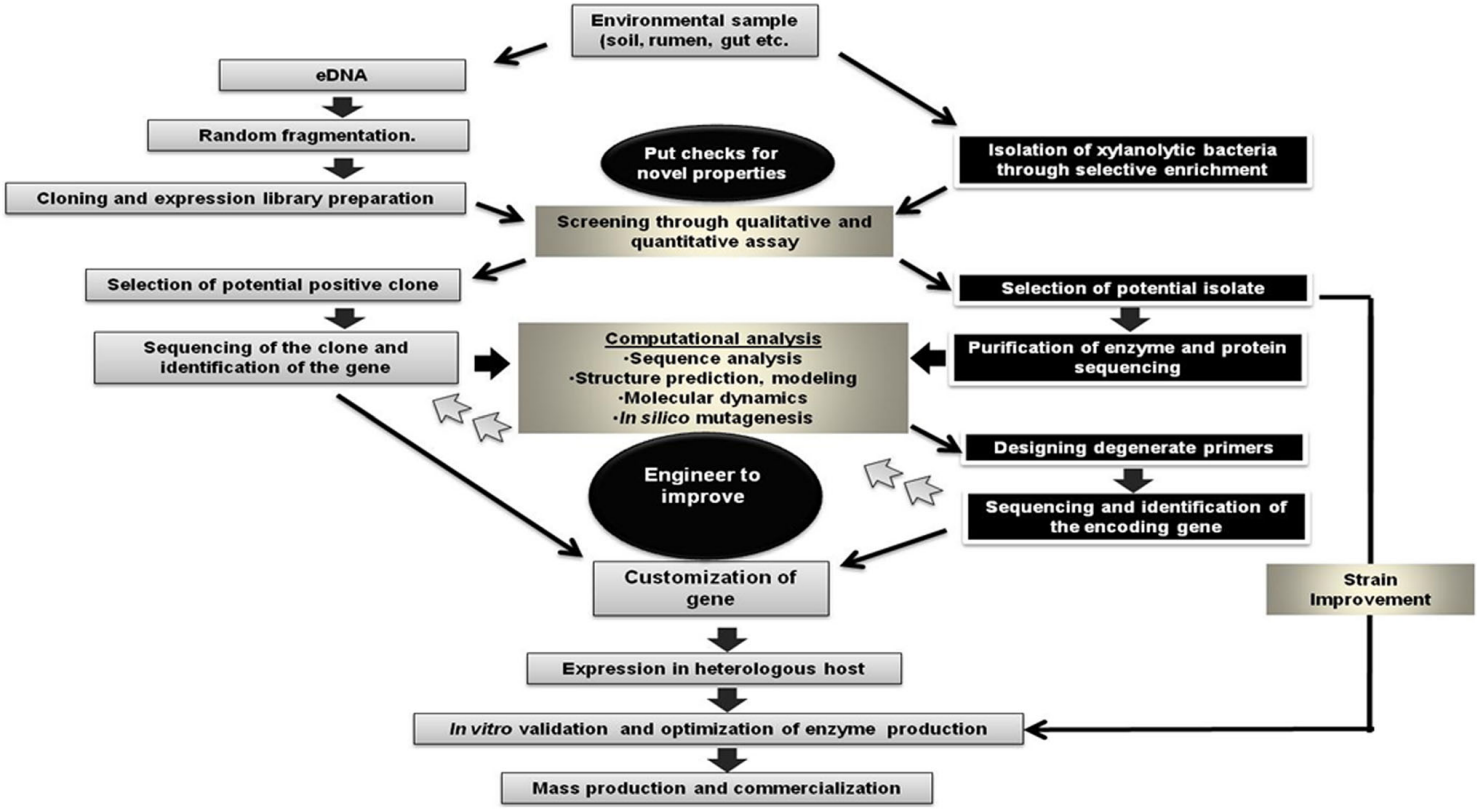
Strategies for designing super-xylanases with novel properties

In many aspects of biotechnological application, bacterial xylanases are advantageous as compared to fungal xylanases. Stability and activity in alkaline range, high thermosatbility, low cellulase activity, greater aggression towards xylans due to high tenacity of carbohydrate domains, and ability to selectively solubilize arabinoxylan make bacterial xylanases amenable to specific industrial application. However, it is very difficult to mine super-xylanases with all these features. Most of the available bacterial xylanases have only few of these properties which make industrial processes to depend much on expensive and hazardous chemical processes. Random mutation through UV or chemical mutagenesis has been a classical way to improve xylanolytic strains for high xylanase activities or inhibiting undesirable activities. However, availability of information on whole genome sequence and metabolic pathways have paved the way for engineering xylanase producing strains suitable for specific industrial application, such as biobleaching of paper pulps, production of biofuel, or XOs. Unlike silencing some properties, adding useful features to xylanase enzymes seems to be more challenging. With the boom of the bioinformatics and public access to whole genome sequences, now, it is possible to design xylanases with industrially desirable features. The techniques used in drug designing like in-silico mutagenesis or simulation of molecular dynamics can be very helpful for designing super xylanases as well as engineering superbugs with multiple catalytic activity. Many of the industrial applications, such as biofuel production, need an array of enzymatic activity for biomass deconstruction. Engineering fusion xylanases with cellulolytic and lignolytic activity may be the choice of the future for biofuel production. However, before going into the advanced techniques, the basic research on isolation of xylanolytic bacteria with novel/high yielding xylanases remains the foremost challenge. Figure 2 presents a comprehensive summary of the probable strategies for designing or engineering super-xylanases. Selection of ecological niche and group of target bacteria is very crucial and can be guided by the genomic information on the abundance of genes responsible for xylanase activities. In the present era of molecular biology and biotechnology, it is not very far to have multi-speciality superxylanases from bacteria which will be better than the best fungal xylanases.

## Conclusion

Increased awareness about environmental pollution by chemical operations in many industries paved a way for usage of more energy efficient, economically viable, and environmental friendly means, such as enzymes. Xylanases are known to have many applications in various industrial processes. Till now, most of the known xylanases are obtained from fungi. Bacterial xylanases offer more competitive and milder operating conditions that put them as a quick substitution for fungal xylanases. Thermo and alkali stable bacterial xylanases have been studied from many bacteria. This gives us a fair idea about genomics and their biochemistry. Modern techniques have further simplified the screening and identification procedures for xylanases. Combination of various methods would help further speed up the research and understanding of bacterial xylanases, so as to have an efficient enzyme to satisfy the want for environmental protection. An in-depth understanding of biology and biochemistry of bacterial xylanases will offer a better scope to search or tailor highly efficient xylanases for effective utilization for industrial purposes.
